# Basic pharmacological mechanisms and clinical evidence of the efficacy of hochuekkito against infectious diseases and its potential for use against COVID‐19

**DOI:** 10.1002/tkm2.1264

**Published:** 2020-12-02

**Authors:** Shin Takayama, Akiko Kikuchi, Toshiaki Makino, Mosaburo Kainuma, Takao Namiki, Takashi Ito

**Affiliations:** ^1^ Department of Kampo Medicine Tohoku University Hospital Sendai Japan; ^2^ Department of Education and Support for Regional Medicine Tohoku University Hospital Sendai Japan; ^3^ Department of Kampo and Integrative Medicine Tohoku University Graduate School of Medicine Sendai Japan; ^4^ Department of Pharmacognosy Graduate School of Pharmaceutical Sciences, Nagoya City University Nagoya Japan; ^5^ Community Medicine Education Unit Graduate School of Medical Science, Kyushu University Fukuoka Japan; ^6^ Department of Japanese‐Oriental (Kampo) Medicine Graduate School of Medicine, Chiba University Chiba Japan; ^7^ Akashi Clinic Kanda Tokyo Japan

**Keywords:** COVID‐19, hochuekkito (hochu‐ekki‐to), immunity, Kampo medicine, viral infection

## Abstract

**Background:**

Hochuekkito extract (HET) has multiple effects through the digestive and immune systems, including for acute viral infection and chronic inflammation. We review basic pharmacological and clinical researches of HET and discuss the effects of HET against the pandemic COVID‐19.

**Methods:**

We reviewed pharmacological studies from 1996 to 30 April 2020 that used experimental animals orally treated with HET and randomized controlled trials (RCTs) from 2000 to 30 April 2020.

**Results:**

Altogether, 64 pharmacological studies reported immuno‐stimulatory effects against infection and cancer, immuno‐modulative effects against allergy and some inflammatory diseases, and ameliorating effects against exhaustion and frailty. Nine RCTs showed improvement of pulmonary *Mycobacterium avium* complex disease on chest X‐ray; improved systemic inflammation, nutrition, and quality of life of patients with chronic obstructive pulmonary disease and a decrease in the number getting common cold and exacerbations; reduction of soluble interleukin‐2 receptor and the serum cortisol concentration of postoperative patients; a reduction of the incidence of inflammatory complications and C‐reactive protein elevation after cerebrovascular disease; a reduction of the volume of steroid and tacrolimus during the treatment of atopic dermatitis; a healing effect for intractable chronic wounds; improvement of the physical status of elderly weak patients; and improvement of the fatigue level of cancer patients.

**Conclusion:**

CODIV‐19 is characterized by high risk for the aged and people with other disease complications, cytokine hyperactivity in the severe stage, and sequelae in the recovery stage. Considering the immune‐stimulative/modulative effects of HET on inflammatory conditions and against exhaustion and frailty, it may be useful for prevention, treatment, and recovery from COVID‐19.

## INTRODUCTION

Human history is also the history of fighting infectious diseases. This was also the situation in ancient East Asia. In the traditional medicine of East Asia, *Shanghanlun* (傷寒論) was written as a treatment manual for acute febrile infections around the 3rd century A.D. Since then, treatments have been taken from it and improved for novel epidemics.

At present, the novel coronavirus disease (COVID)‐19 has become pandemic [1], and there is an urgent need to develop treatments for it. In China, several herbal medicines have been used to treat COVID‐19 and have been reported to successfully have some effect on COVID‐19 [2, 3]. However, these reports are not of high level, such as randomized control trials (RCTs). The infectious pattern of COVID‐19 is extremely complex. It is problematic that an affected person can infect other people about 48 h before the onset of symptoms; thus we cannot predict who will infect others, making not only treatment but preventive measures for such infections critical. COVID‐19 prevention measures require the development of vaccines along with standard measures such as hand washing. Use of vaccine is critical, but this will take time to develop. Furthermore, because this novel coronavirus may mutate, additional therapies may be required.

Hochuekkito (hochu‐ekki‐to, bu‐zhongzhong‐yi‐qi‐tang, 補中益気湯: decoction), the focus of this article, is a drug with complex effects that has both treatment and prophylactic effects and improves the harmful effect of mental stress on the body. Its mechanism has not been sufficiently scientifically clarified because it is a mixture of crude drugs. In Japan, the industrially produced Kampo formulation of hochuekkito extract preparation (HET) is commonly used for research, and its quality is constant and well standardized [4, 5]. Kampo medicines are widely used for therapeutic purposes because of their portability and ease of administration. In 1967, HET was permitted by the Japanese national health insurance system for the treatment of several conditions and diseases, such as reduced digestive function, anorexia, gastroptosis, and visceral prolapse.

Most studies about hochuekkito use HET instead of a hochuekkito decoction formula because its quality and ingredients are stable. The crude drugs used in HET are standardized in the Japanese Pharmacopeia [4, 6], and the contents of the crude drugs and their production process have been authorized by the Medicines Regulatory Agency of the Japanese Government [7]. Therefore, for use in medical research, HET can be used without definition of each of the crude drugs and doses [4, 5]. The amount of crude drug used in Japan is considerably less than one‐third that used in China. When used in Japanese amounts, side effects are clinically reported at a lower rate than for Western drugs in Japan [8, 9]. It is important to be cognizant of the different research conditions of Japan and China.

In this report, we briefly introduce the history and traditional role of HET, then the development of research evidence for HET over the past few years. We summarize experimental and clinical studies, especially for respiratory and immunological diseases, then discuss COVID‐19‐related prevention, treatment, and recovery.

### History of hochuekkito

Hochuekkito is an herbal drug first described by Li Gao (1180–1251) in his medical books ‘Clarifying Doubt about Damage from Internal and External Causes’ and ‘Treatise on Spleen and Stomach (the Theoretical Relation between Disease and Digestive Function)’ [10]. It is used to supplement the functions of the digestive system, the spleen [TM; meaning in traditional medicine], and stomach [TM], and acts to increase vital energy. It is also known as ‘Io‐to (医王湯)’ which means the king of Kampo (Japanese traditional) medicines. The following experience greatly influenced Li Gao and led to his formulation of hochuekkito. His city was surrounded by the Mongolian army. Seeing mass death and finding that the usual treatment for damage from external factors, such as infectious disease, did not always work and that many people died despite his efforts to treat them, he recognized the importance of internal damage when the digestive system is injured due to mental or physical fatigue. The concepts that hochuekkito is based on are that people live through the energy that is contained in food and drink and that the work of the digestive system is the basis of human activity. However, if something interferes with proper eating or drinking, the digestive system is damaged and various diseases begin to develop. Li Gao called this ‘internal damage’ and postulated that it could be cured with a warming, sweet‐tasting medicine that supplements the vital energy of the spleen [TM] and stomach [TM], thus raising the vital energy and extinguishing the heat of the body. This led to his creation of hochuekkito.

Hochuekkito consists of a combination of 10 crude drugs: Astragalus Root, Ginseng, Atractylodes Rhizome (or Atractylodes Lancea Rhizome), Japanese Angelica Root, Citrus Unshiu Peel, Ginger, Jujube, Bupleurum Root, Glycyrrhiza, and Cimicifuga Rhizome [5, 6]. Ginseng, Astragalus Root, Bupleurum Root, and Cimicifuga Rhizome play especially important roles. Ginseng promotes a healthy stomach and is a tonic that is useful in the treatment of anorexia, malaise, and diarrhea by improving a decline in metabolism due to gastrointestinal weakness. Astragalus Root suppresses abnormal sweating and improves fluid [TM] toxicity in body surface water poisoning by diuresis [11]. It also acts as a tonic and is used for the treatment of weak people and malnutrition. Kampo formulations (hoZAI in Japanese) that contain Ginseng and Astragalus Root (ninJIN and oGI in Japanese, respectively) are ‘*JIN‐GI‐ZAI*’. ‘*JIN‐GI‐ZAI*’ clinically improves functions related to food digestion and absorption to activate systemic nutritional status, restores biological defense functions, and promotes healing. Bupleurum Root and Cimicifuga Rhizome have antipyretic effects, and it is presumed that they have the effect of raising qi. Through these comprehensive actions, hochuekkito assists in the action of the digestive system and enhances physical strength. Hochuekkito was created with the qi‐tonifying properties of Ginseng and Astragalus Root, which were first noted in *Xiaopingfang* (小品方), published in 5th century [12], and applies the theory of Zhang Yuan‐Su (1151–1234, Li Gao's teacher) who wrote ‘*Yixueqiyuan* (医学啓源, Revelation of Medicine)’ [13] and emphasized the combination of Bupleurum Root and Cimicifuga Rhizome.

Hochuekkito has been widely used in Japan since the 16th century, and there was a huge surge in writings about it during the Edo period. Tokaku Wada said, ‘The purpose of hochuekkito itself was to assemble the pain and discomfort and resistance found on both sides of the lower part of the heart. It was a modified formulation of shosaikoto (xiao‐chai‐fu‐tang, 小柴胡湯)’. Sohaku Asada described its proper use in this way: ‘use it for those who have the deficiency pattern of shosaikoto’. The description of patients who need hochuekkito by Gensen Tsuda in the 18th century, during the Edo period, is famous for its eight *kuketsu* (traditional keys of treatment, clinical pearls): (i) person feeling tired in the limbs; (ii) voice is small to hear; (iii) the eyes are powerless; (iv) white drops are seen in the mouth; (v) there is a loss of taste; (vi) a preference for hot food and drink; (vii) palpating the abdomen near the umbilicus; and (viii) the pulse is paradoxically large and powerless. It was said that if one or two of these eight symptoms were present, hochuekkito would be the proper prescription [10].

**Figure 1:**
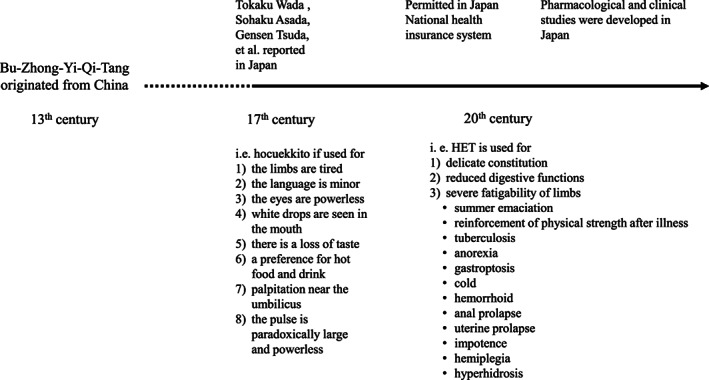
History and development of hochuekkito in Japan.

Figure 1 shows the history and adaptation of hochuekkito. The original concept was described by Li Gao in the 13th century, as previously described. It was applied for various conditions in the Edo period by Tokaku Wada, Sohaku Asada, GensenTsuda, and others.

## PHARMACOLOGICAL STUDIES ON HET

We searched for all non‐human studies using HET published after 1996 and registered in the PubMed and Ichu‐shi databases, and hit 117 articles. Then, we chose and reviewed 53 articles that used experimental animals orally treated with not‐excessive dosages (less than 25 times the human daily dosage relative to body weight) in order to translate these basic results for use in clinical medicine. We did not cover the experimental results of HET studies that used cultured cells because the active ingredients of HET for immune‐stimulatory and immune‐modulative effects are polysaccharides [14, 15]. These high‐molecular‐weight compounds are not absorbed from the intestinal lumen into the circulation, but in their original forms act directly on body tissue cells. However, these compounds can stimulate immune cells in intestinal epithelium to exhibit immune‐stimulatory and immune‐modulative effects throughout the body.

The pharmacological research articles reviewed were published from 1996 to 30 April 2020. They include only articles written after the review by Song in 1996 [16, 17]. In Table 1, we summarize the pharmacological effects of HET that support its clinical effectiveness for various diseases.

**Table 1:** Summary of pharmacological studies of HET in animal experiments

Effects	Dosage of HET^†^	Pharmacological events
Immuno‐stimulatory effects against infection and cancer	10‐fold, HET ethanol insoluble fraction, 7 days	Nitric oxide production from peritoneal macrophage in mice increased in normal mice [18]
	7‐ or 10‐fold, 1 or 32 weeks	Natural killer (NK) activity of spleen cells increased in mice (1 week). NK1.1^+^ cells and CD4^+^/CD8^+^ cells in spleen cells were increased in mice (32 weeks) [19]
	20‐fold, 14 weeks	Number of CD3^+^ and NK1.1^+^ cells in spleen and the production of antigen‐specific antibody from spleen cells sensitized with sheep red blood cells increased in aged mice [20]
	10‐ and 20‐fold, 4 days	Immune deficiency was induced by prednisolone injection in mice. Survival rate after the injection of candida increased [21]
	4‐ and 8‐fold, 2–14 days	Immune deficiency was induced by cyclophosphamide injection in mice. Number of blood monocytes and neutrophils in spleen cells, and the production of IL‐3, GM‐CSF, and IFN‐γ from spleen cells was improved [22]
	5‐ and 10‐fold, 10 days	Immune deficiency was induced by mitomycin C injection in mice. Weight of spleen, NK activity of spleen cells, number of granulocytes and macrophages in bone marrow cells increased. Survival rate after herpes virus infection increased [23]
	10‐fold, 12 days	Immune deficiency was induced by restraint stress in mice. Serum concentration of corticosterone and IL‐12 recovered. Number of cells in spleen and lymph node, and NK activity of spleen cells increased. Tumor size decreased in mice subcutaneously injected with melanoma [24]
	8‐fold, 7 days	Immune deficiency was induced by restraint stress in mice. Serum concentration of corticosterone and number of macrophages and bactericidal activity in peritoneal cells, and the production of IFN‐γ from spleen cells recovered in mice injected with *Listeria monocytogenes* [25]
	4‐fold, 8 times in 24 days	Virulent *Salmonella enteritidis* strain was injected in mice subcutaneously inoculated with lymphoma. Survival rate and IFN‐γ production from spleen cells increased [26]
	8‐fold, 7 days	*Listeria monocytogenes* was orally treated in mice. Number of *Listeria* in liver and Peyer's patch decreased. Bactericidal effect of collected peritoneal macrophages increased. Intestinal epithelia lymphocytes produced higher IFN‐γ [27]
	8‐fold, 7 days	*Listeria monocytogenes* was injected in mice. Number of infected *Listeria* in spleen and liver decreased. Spleen cells stimulated by antigen produced higher IFN‐γ. Number of macrophages and MHC class II cells in peritoneal cells increased [28]
	10‐fold, 3 weeks	*Helicobacter pylori* was orally treated in mice. Number of bacteria in gastric mucosa reduced [29]
	20‐fold, 4 days	Methicillin‐resistant *Staphylococcus aureus* (MRSA) was innoculated into murine nostrils. Number of bacteria in nasal fluids decreased. Proliferation of isolated spleen cells increased [30]
	0.1–10‐fold, 11 days	Influenza virus was intranasally infected in mice. Survival rate and IFN level in BALF increased. Virus titer, IL‐1α, IL‐6, and GM‐CSF levels in BALF reduced [31]
	7‐ or 10‐fold, 2 weeks	Influenza virus was intranasally infected in mice. Survival rate and IFN‐α level in BALF, IFN‐α, β, defensins, and GM‐CSF levels in lung tissue increased [32, 33]
	10‐fold, 2 weeks	Influenza virus was infected intranasally in SAMP‐1 mice. TNF‐α, IL‐1β and IL‐12 levels in lung, phagocytosis activity of alveolar macrophage increased [34]
	3–9‐fold, 2 weeks	Inactivated influenza virus was inoculated into cyclophosphamide‐treated mice. Antigen‐specific IgA and IgG in nasal wash, BALF, and serum increased [35]
	16‐fold, every 2 days in 7 days	Murine cytomegalovirus was injected in mice. Number of neutrophils and NK cells in spleen and liver, NK activity of spleen cells, serum IFN‐α/β level, phagocytosis activity of macrophage in intraperitoneal cells increased, and the titer of virus in spleen reduced [36]
	13‐ or 20‐fold, 15 days	Murine red blood cells infected with malaria parasite *Plasmodium chabaudi* was injected in mice. Survival rate, serum IFN‐γ level increased, and serum IL‐12 decreased [37]
	2.5‐ and 5‐fold, 6–16 days	Fibrosarcoma cells were subcutaneously inoculated in mice. Tumor size and number of cells in lymph node reduced [38]
	10‐fold, 21 days	Colon carcinoma cells were injected into portal vein in mice. Number of tumor colonies in liver reduced [39]
	4‐fold, 18 or 25 weeks	Carcinogenesis was induced by 12‐*O*‐tetradecanoylphorbol‐13‐acetate in 7,12‐dimethylbenz[a]antracene painting or 4‐nitroquinoline‐1‐oxide subcutaneous injection in mice. Number of tumor colonies in skin and lung reduced [40]
	1‐fold, 2 and 30 weeks	Endometrial cancer was induced by ovariectomy, oral treatment of estradiol, and *N*‐methyl‐*N*‐nitrosourea injection into uterine tract in mice. mRNA expressions of c‐jun, TNF‐α, and ER‐α and ‐β in uterus reduced. Histopathological symptoms improved [41]
	7‐ or 10‐fold, 18 weeks	Biliary cancer was induced by choledochojejunostomy and *N*‐nitrosobis (2‐oxopropyl) amine subcutaneous injection in hamster. Serum ALT and aspartate aminotransferase (AST) levels, number of individuals with cancer appearing, and the proliferation of biliary epithelial cells reduced [42]
Immuno‐modulative effects against allergy and inflammatory diseases	8‐fold, 4 weeks	Allergy was induced by sensitization with DNP‐KLH in mice. Serum antigen‐specific IgE level, the production of IgG_1_, IL‐2, and IL‐4 from spleen cells, number of eosinophils and CD4^+^ cells in intraperitoneal cells, the production of IL‐4 and IL‐5 from intraperitoneal cells, and delayed hypersensitivity reaction decreased [43, 44]
	20‐fold, 25 days	Allergy was induced by TNCB painting onto ear in mice. Edema, serum IL‐4, IL‐5 and IgG_2a_ levels, antigen‐specific IgE, IgG_1_ and IgG_2a_ levels, IL‐4 level in ear reduced [45]
	10‐fold, 12 days	Allergy was induced by TNCB painting onto ear of NC/Jic atopic mice. Scratching behavior level and degranulation from mast cells in ear tissue reduced [46]
	1‐ and 10‐fold, single time	Allergy was induced by ovalbumin injection in mice. Serum antigen‐specific IgE level, the proliferation and IL‐4 production in spleen cells induced with antigen, and the production of histamine from eosinophils in blood decreased [47]
	8‐fold, 7 days	Allergy was induced by ovalbumin injection in mice. Serum antigen‐specific IgE and IgG_1_ levels decreased. CD4^+^ cells in Peyer's patch and the production of IL‐12 from Peyer's patch cells increased [48]
	8‐fold, single time or 8 days	Allergy was induced by ovalbumin inhalation in mice. Number of neutrophils, IL‐4 and IL‐5 levels in BALF and serum IgE and IgG_1_ levels reduced. Serum IgG_2a_ level increased. Proliferation and IL‐4 production in T cells separated from spleen induced by antigen reduced, and IFN‐γ production increased [49]
	8‐fold, 2 weeks	Allergy was induced by ovalbumin in germ‐free mice. Serum antigen‐specific IgG_1_ level reduced. IFN‐γ production and Th1/Th2 ratio in spleen cells induced with antigen increased [50]
	2‐ and 7‐fold, 14 days	Allergy was induced by ovalbumin in NC/Jic mice. Serum ALT level and production of IL‐4, IL‐6 and TNF‐α from liver cells reduced [51]
	2‐ and 7‐fold, 14 days	Allergy was induced by ovalbumin in NC/Jic mice. Serum ALT level, number of multinuclear cells and CD4^+^ cells in liver, the production of IL‐4, IL‐6 and TNF‐α from liver cells, and edema in small intestine reduced [52]
	1–25‐fold, 3 weeks	Rheumatism was induced by subcutaneous injection of type‐II collagen and complete Freund's adjuvant in mice. Score of arthritis, serum antigen‐specific IgG level reduced. Number of CD3^+^CD4^+^ cells, CD3^+^CD8^+^ cells, CD8^+^T cells, and CD3^−^B220^+^ in blood, axillary and inguinal lymph nodes improved [53, 54]
	1‐ and 3‐fold, 23 days	Autoimmune encephalomyelitis was induced by subcutaneous injection of MBP and complete Freund's adjuvant combined with subcutaneous injection of MBP at ear in rats. Encephalomyelitis score, ear edema, and serum anti‐MBP antibody titer decreased [55]
	7‐fold, 1–10 days	Intestinal mucitis was injected by methotrexate injection in mice. TNF‐α, CCL‐25, keratinocyte growth factor, IL‐1β, single immunoglobulin and toll‐IL‐1 receptor domain, IL‐1 receptor related kinase 3, CYLD lysine 63 deubiquitinase, TNF‐α‐induced protein 3 levels and tissue injury in jejunum, and CCL20 level in Peyer's patch recovered [56, 57]
	25‐fold, 8 days	CBA mice underwent transplantation of a C57BL/6 (H2b) mouse heart. Median survival time prolonged [58]
	7‐fold, 19 weeks	Hepatitis was induced by oral treatment with thioacetamide in rats. Serum γ‐globulin reduced [59]
	1‐ and 2‐fold, 58 days, 5‐times a week	Hepatitis was induced by the injection of pig serum in rats. Hydroxyproline and IL‐13 levels in liver, and serum transforming growth factor (TGF)‐β1 level reduced [60]
	10‐fold, 8 weeks or 35 days	Number of neutrophils and macrophages in BALF and keratinocyte chemoattractant level in serum reduced in murine lung injury model induced by nasal injection of LPS) [61]. Histological score, hydroxyproline levels and IL‐5/IFN‐γ mRNA expression ratio in lung tissue improved in murine lung injury model induced by bleomycin inhalation [62]
	23‐fold, 1 week	Ultraviolet light was applied to hairless mice. Transepidermal water loss, water content, catalase activity, and carbonylated protein content in stratum corneum improved [63]
	1.5‐ and 3‐fold, 14 days	Writhing behavior induced by acetic acid injection in mice improved [64, 65]
Ameliorating effects against exhaustion and frailty	1‐fold, 14 days	Locomotor activity increased in mice [66]
	3‐ or 5‐fold, 30 days	CFS was induced by the injection of *Brucella abortus* antigen in mice. Spontaneous locomotor activity and IFN‐γ mRNA expression in spleen increased [67]
	5‐fold, 4 weeks	CFS was induced by the injection of *Brucella abortus* antigen in mice. Weight of thymus and NK activity in spleen cells increased. Weight of spleen reduced [68, 69]
	1.5‐ and 3‐fold, 14 days	Depression was induced by water immersion and restraint stress in mice. Locomotor activity improved [70]
	10‐ and 20‐fold, 4 weeks	Epilepsia was induced by cobalt application to the cerebral cortex in rats. Neuron loss in CA1 area of hippocampus improved [71]
	12‐fold, 4 days	Serotonin 2C receptor mRNA expression in rat brain increased [72]
Diabetes	0.5‐fold, 4 weeks	Diabetes was induced by streptozotocin injection in mice. TNF‐α production from alveolar macrophage improved [73]
Osteoporosis	5‐fold, 8 weeks	Osteoporosis was induced by ovariectomy in rats. Serum alkaline phosphatase activity, estradiol, progesterone and dehydroepiandrosterone sulfate levels, bone mineral density values improved [74, 75, 76]
	8‐fold, 8 weeks	Osteoporosis was induced by subcutaneous injection of gonadotropin‐releasing hormone in rats. Serum estradiol level and bone mineral density values improved [77]
Microgravity environment	10‐fold, 3 days	Microgravity environment for 10 s was applied to mice injected with saline. Reduction of body weight improved. Serum antidiuretic hormone, phosphorus, and estradiol levels improved [78, 79]
Condition of enteric bacteria	7‐ or 10‐fold, 14 days	*Escherichia coli* level in feces increased in mice [80]

^†^ Ratio to human daily dosage of HET as body weight conversion and the duration of the oral administration.

HET, hochuekkito extract; NK, natural killer; IL, interleukin; GM‐CSF, granulocyte macrophage colony‐stimulating factor; IFN, interferon; MHC, major histocompatibility complex; BALF, bronchoalveolar fluid; SAMP, senescence‐accelerated mouse prone; TNF, tumor necrosis factor; ER, estrogen receptor; DNP‐KLH, 2,4‐dinitrophenol keyhole limpet hemocyanin; TNCB, 2,4,6‐trinitrochlorobenzene; ALT, alanine aminotransferase; MBP, myelin basic protein; CCL, chemokine ligand; CYLD, cylindromatosis; LPS, lipopolysaccharide; CFS, chronic fatigue syndrome.

### Immuno‐stimulatory effects against infection and cancer

**Figure 2:**
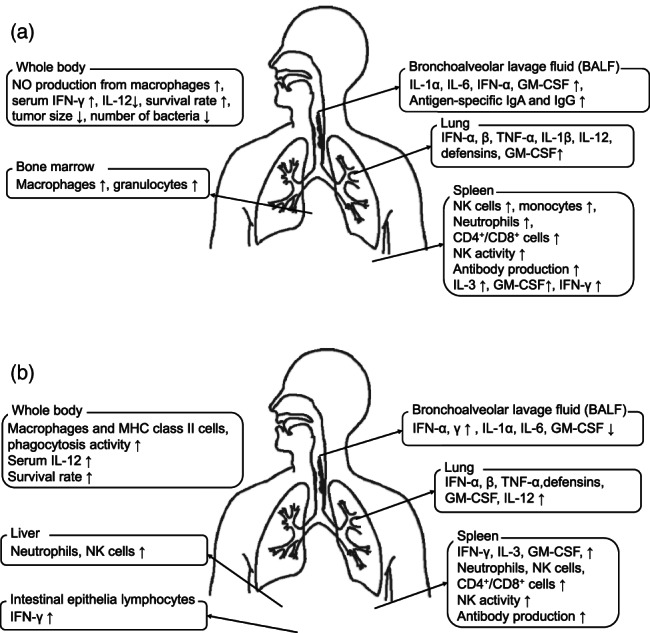
Schema of the immuno‐stimulatory (a) and immuno‐modulative (b) effects of HET against infection.

There are several reports about the immuno‐stimulatory effects of HET (Fig. 2a). In two studies, HET augmented cell functions related to the innate immune system of normal animals [18, 19]. In animal models of immune deficiency by age [20], steroids [21], cyclophosphamide [22], mitomycin C [23], and restraint stress [24,25], HET improved reduced immune function by both innate and acquired immunity. Related to these immuno‐stimulatory effects, HET exhibited a preventive effect from infection by *Salmonella enteritidis* [26], *Listeria monocytogenes* [27, 28], *Helicobacter pylori* [29], methicillin‐resistant *Staphylococcus aureus* (MRSA) [30], influenza virus [31, 32, 33, 34, 35], cytomegalovirus [36], and *Plasmodium chabaudi* [37]. HET also exhibited a protective effect on cancer caused by inoculation of cancer cells [38, 39] or carcinogenic substances [40, 41, 42].

### Immuno‐modulative effects against allergy, and some inflammatory diseases

HET also has been shown to be effective against diseases related to the immune system (Fig. 2b). HET exhibited a suppressive effect on allergic diseases caused by hapten [43, 44, 45, 46] and ovalbumin (OVA) [47, 48, 49, 50, 51, 52]. HET improved rheumatism [53, 54], autoimmune encephalomyelitis [55], intestinal mucositis [56, 57], transplantation rejection [58], hepatitis [59, 60], pulmonary disease [61, 62], dermatitis [63], and pain induced by inflammation [64, 65].

### Ameliorating effects against exhaustion and frailty

HET was shown to promote locomotor activity by normal mice [66] and in a chronic fatigue syndrome (CFS) model induced by the injection of *Brucella abortus* antigen [67, 68, 69]. HET exhibited effectiveness in animal models of depression [70] and epilepsia [71]. Its effects on serotonin and the nervous system may contribute to these effects [72].

### Miscellaneous

There are several reports about the pharmacological effects of HET in animal models of diabetes [73], osteoporosis [74, 75, 76, 77], and a microgravity environment [78, 79]. HET has been shown to modulate the condition of enteric bacteria [80].

## CLINICAL STUDIES ON HET

Although few clinical studies have investigated the efficacy of HET, we summarize the reports made to date. The keywords ‘hochuekkito’, restricted RCT, systematic review, and meta‐analysis were used to search the PubMed and Ichushi databases for clinical research articles written in English or Japanese. We selected articles published from 2000 to 30 April 2020, because the designs and evaluation methods of studies regarding Kampo medicine before 2000 were considered insufficient by modern research evaluation standards. Protocol articles and articles published in journals for commercial purposes that explained the same content as in previous reports were excluded. Consequently, we found no systematic review or meta‐analysis focused on an RCT of HET. As shown in Table 2, the data of nine RCTs were available for inclusion. We herein introduce the principal reports with evidence of the influence of HET on inflammation and nutrition.

**Table 2:** Randomized controlled trials of published from 2000 to 30 April 2020

Publication year	Authors	Study type	Subject (*n*)Kampo group/control group	Age (years old)Kampo group/control group	Disease/symptom	Kampo group	Comparison	Outcome	Adverse events	Reference
2019	Akita *et al*.	RCT	18 (9/9)	50 ± 10/54 ± 16	Chronic wounds refractory to conventional therapies	HET + conventional therapies	Conventional therapies	At 12 weeks, wound healing progressed in all nine patients in HET group, whereas only three patients in the control group recovered. In HET group, the total wound severity scores decreased significantly at 8 weeks and later	No	[81]
2017	Fukumura *et al*.	RCT	28 (11/17)	74.6 ± 7.1/78.8±8.6	Undergoing rehabilitation for hemiplegia after cerebrovascular disease	HET	No Kampo	The incidence of inflammatory complications was significantly lower in HET group. There was no significant difference in the changes of functional independence measure score between the two groups	5 in HET group, 10 (3 severe) in the control group. No severe adverse events in HET group	[82]
2014	Enomoto *et al*.	quasi‐RCT	18 (9/9)	70 (44–80)/70(59–83)	Progressed pulmonary *Mycobacterium avium* complex disease	HET + preceding treatment	Continue the preceding treatment	None of the patients achieved sputum conversion. Radiological disease control was more frequently observed in HET group than in the control group. Patients in HET group tended to increase in body weight and serum albumin level	No adverse events greater than grade 3	[83]
2010	Kobayashi *et al*.	DBRCT	84 (40/44) completed participants: 77(37/40)	27.3/27.5	Kikyo (delicate constitution) with atopic dermatitis	HET + ordinary treatments	Placebo + ordinary treatments	The total equivalent amounts of topical agents were significantly lower in HET group. The aggravated rate was significantly lower in HET group than in the placebo group	Only mild adverse events were noted in both groups without statistical difference	[84]
2010	Jeong *et al*.	RCT	40 (20/20)	49.4 ± 10.8/53.4 ± 8.0	Cancer‐related fatigue	HET	Waiting list control group	HET group showed statistically significant improvements in fatigue level assessed by the Visual Analogue Scale of Global Fatigue and in scales which measure the QOLof cancer patients	No serious adverse effects	[85]
2009	Tatsumi *et al*.	RCT	71 (34/37)	Elderly	Moderate to severe chronic obstructive pulmonary disease	HET + conventional therapy	Conventional therapy	In HET group, body weight significantly increased for 6 months, the patients' QOL score improved. The number of common colds and acute exacerbations was significantly lower. CRP, TNF‐α, IL‐6, and serum pre‐albumin increased.	No	[86]
2007	Shinozuka *et al*.	RCT	35 (17/18)	73 ± 1	Chronic obstructive pulmonary disease	HET+ bronchodilators	Bronchodilators	In HET group, serum CRP and TNFα significantly decreased, serum albumin level significantly increased	N/A	[87]
2006	Saito *et al*.	RCT	48 (22/26)	64.0 ± 2.7/67.8 ± 1.8	Postoperative patients with stomach or colon cancer	HET administration for 7 days before operation	No Kampo	The value of soluble IL‐2 receptor just before the surgery, serum cortisol concentration on the 1st day after the surgery, body temperature after the surgery were significantly decreased in patients in HET group compared to those in control group	No adverse events due to HET	[88]
2005	Satoh *et al*	DBRCT	15	78.4 ± 7.8	Elderly patients with weakness	HET	Placebo	The physical component summary of the SF‐36 analysis significantly improved in HET group. Four components out of six improved in HET group in the POMS analysis. The population of CD3^+^ peripheral lymphocytes and CD3^+^/CD4^+^ cells increased in HET group	No	[89]

HET, ethical formulation of hochuekkito extract preparations; RCT, randomized controlled trial; QOL, quality of life; DBRCT, double‐blind randomized controlled trial; CRP, C‐reactive protein; TNF, tumor necrosis factor; IL, interleukin; SF‐36, Short Form 36 Health Survey; POMS, Profile of Mood States; N/A, not available.

### The effects on infection, inflammation, and allergy

As shown above, an immune‐stimulative and modulative effect has been reported in experimental studies. From this point of view, infection (immune‐stimulation), inflammatory condition (immuno‐modulation), and allergy (immuno‐modulation) have been reported in clinical studies. The effect on nutrition as it relates to immune conditions has been reported in some articles.

#### Infection (immune‐stimulation and nutrition)

##### Pulmonary Mycobacterium avium complex disease

A pilot, open‐label, quasi‐randomized, controlled trial investigated whether HET has benefits for and how it is tolerated by patients with progressed pulmonary *Mycobacterium avium* complex disease [83]. The authors divided 18 patients into a baseline treatment group and a HET administered preceding treatment group. After the 24‐week treatment period, no patient achieved sputum conversion. The HET group tended to have improved chest X‐ray images compared to the control group. The body weight and serum albumin level of the HET group tended to be increased compared with those of the control group. Because this study had a small size, there was no apparent difference between the control and HET groups. Nevertheless, these results suggested that HET may have a favorable impact on nutritional status and beneficial effects for patients with progressed pulmonary *Mycobacterium avium* complex disease. Further placebo‐controlled and large‐scale studies will be necessary to clarify the role of HET.

#### Inflammatory condition (immuno‐modulation and nutrition)

##### Chronic obstructive pulmonary disease (COPD)

In 2007, Shinozuka *et al*. reported that HET significantly reduced serum C‐reactive protein (CRP) and TNF‐α levels and increased the serum albumin level of patients with COPD [87]. This was also shown in the RCT reported by Tatsumi *et al*. in 2009 [86]. It demonstrated that HET reduces the number of common colds and acute exacerbation episodes of patients with COPD. Furthermore, HET decreased the serum CRP, TNF‐α, and IL‐6 level and increased the serum pre‐albumin level. The body weight of participants in the HET group was significantly increased, and the St. George's Respiratory Questionnaire score, which evaluates the quality of life of COPD patients, was significantly improved in the HET group. These reports indicated that HET improved the systemic inflammation, nutrition, and quality of life of COPD patients, decreasing the number of patients getting common cold and having exacerbations. The immunological and anti‐inflammatory effects of HET may have contributed to these results.

##### Systemic inflammatory response syndrome (SIRS) for postoperative patients with stomach or colon cancer

Saito *et al*. conducted a multicenter RCT to investigate the effect of seven‐day preoperative administration of HET on postoperative SIRS for patients undergoing gastrectomy or colectomy [88]. In the HET group, the value of soluble IL‐2 receptor just before surgery tended to be low and the serum cortisol concentration on the first day after the surgery, body temperature, and the usage of second‐line antibiotics after the surgery were significantly decreased compared to those of the control group. There were no differences between the groups in the number of white blood cells, the level of CRP, or days of postoperative hospitalization. The authors speculated that the anti‐inflammatory and immune‐modulative effect of HET may reduce cytokines produced by surgical stress and prevent SIRS and postoperative infection.

##### Inflammatory complications after cerebrovascular disease

Fukumura *et al*. [82] reported that for patients undergoing rehabilitation for hemiplegia after cerebrovascular disease, HET reduced the incidence of inflammatory complications such as pneumonia, cholecystitis, cystitis, and other infectious diseases for which serum CRP was elevated 10 mg/mL and over. There was no significant difference in the change of the functional independence measure score between a HET plus rehabilitation group and a rehabilitation‐alone group. These results suggest that HET can be useful for preventing inflammatory complications during rehabilitations for severe sequelae of cerebrovascular disease.

##### Allergy in atopic dermatitis (AD)

Kobayashi *et al*. conducted a double‐blind (DB) RCT regarding the effect of HET on the AD of patients with qi deficiency (delicate, easily fatigable, hypersensitive) [84]. The patients taking HET did not show significant improvement of overall skin severity scores compared with a placebo group, but the volume of steroid and tacrolimus was significantly reduced. The prominent efficacy rate increased, but not significantly, in both the HET and placebo groups. Furthermore, the aggravated rate was significantly lower in the HET group. In 2012, they re‐analyzed the data using eczema [90]. HET is usually prescribed for popular/nodular/lichenization eczema rather than moist/eschew eczema. These studies demonstrated that HET is a useful complementary treatment for AD patients with a qi deficient constitution and may reduce the treatment dosage of topical steroids and/or tacrolimus without aggravating AD.

### Ameliorating effects against exhaustion and frailty

As shown above, ameliorating effects against exhaustion and frailty have been reported in experimental studies. Wound repair, fatigue, and weakness have also been reported in clinical studies. The effects on nutrition related to exhaustion and frailty have been reported in some articles.

#### Intractable chronic wounds

Akita *et al*. reported the effect of HET on intractable chronic wounds [81]. They randomly allocated 18 patients to HET and no‐HET administration groups. After 12 weeks, all nine participants in the HET group had an improved DESIGN‐Rating score, which evaluates the severity of the wound, compared with only three participants in the control group. The DESIGN‐Rating scores were analyzed every four weeks. In the HET group, the total value of the DESIGN‐R score gradually decreased, showing a significantly lower value at eight weeks and later compared to the initial value. In the control group, the score did not improve significantly during the study period. Neither CRP nor IL‐6 showed any significant change in either group. But in the HET group, serum albumin was increased at week 12. Thus, the data indicated that the wound‐healing effect of HET was related to the improved nutrition of the patients. Because this report had a small sample size, further study will be necessary to evaluate the effect of HET on healing wounds.

#### Elderly patients with weakness

It is difficult to conduct RCTs of Kampo medicine because of its distinctive diagnostic system, far different from that of conventional medicine, and difficulty in making appropriate placebos due to the smells and tastes of the crude drugs. In 2005, Satoh *et al*. used a unique study design that combined a double‐blind, placebo‐controlled, quasi‐cross‐over study that was HET responder‐restricted [89]. After the run‐in period, only responder elderly participants were randomly allocated into three groups: HET–placebo group, placebo–HET group, and HET–HET group. Each intervention period was six weeks, with a two‐week washout period. The physical component summary of the Short Form 36 Health Survey analysis was significantly improved in the HET‐treated group. Four of the six components (anger–hostility, fatigue, tension–anxiety, confusion) of the Profile of Mood States were improved in the HET group. There were no differences in natural killer (NK) cytolytic activity, IL‐2 production ability, or lymphocyte proliferating activity. The population of CD3^+^ cells and CD3^+^CD4^+^ cells significantly increased in the HET group. These data suggested that HET may improve the quality of life and immunological status of elderly patients with weakness by influencing the immunoreactivity of CD4^+^ or CD3^+^ T lymphocytes.

#### Cancer‐related fatigue

Fatigue is a frequent symptom that degrades the quality of daily activities of living of cancer patients. However, cancer‐related fatigue has been undertreated because of its various causes and multifactorial conditions, such as the cancer itself, adverse effects of therapy, psychological depression, and poor nutrition. In 2010, Jeong *et al*. reported a pilot clinical trial on the efficacy of HET for patients who had cancer‐related fatigue [85]. A total of 40 patients were allocated randomly into a HET group or a waiting list control group. A two‐week administration of HET significantly improved the fatigue level as assessed by the Visual Analogue Scale of Global Fatigue and the results of Functional Assessment of Cancer Therapy‐General, Functional Assessment of Cancer Therapy‐Fatigue, and Trial Outcome Index‐Fatigue. These results suggest that HET may be beneficial for reducing cancer‐related fatigue, which would improve the quality of life of cancer patients.

## DISCUSSION

The research summaries of basic and clinical studies presented here have elucidated the various effects of HET, a Kampo extract formulation made in Japan.

### HET, the decoction of hochuekkito, and bu‐zhong‐yi‐qi‐tang: their differences

Bu‐zhong‐yi‐qi‐tang is a decoction form of the drug. When it is prescribed, crude drugs are added or subtracted from the original formula used in China. It is important to note that the characteristics of bu‐zhong‐yi‐qi‐tang are not the same as those of the substance used in China. In contrast, HET is industrially produced as a uniform product in Japan, and its quality is strictly controlled. Products with the same lot number from the same manufacturer are the same formulation and contain the same ingredients. The composition of the crude drugs used in Japan and China is different, and the dosage of the crude drugs is less than one‐third in Japan, even though the drug may have the same name. This situation is the same for the products and decoctions of Korea. Standardized quality is necessary for research to insure the accuracy of the data, thus the research results for studies of bu‐zhong‐yi‐qi‐tang and hochuekkito done in China and South Korea have been excluded from this report.

### Immune system, inflammation and infection

**Figure 3:**
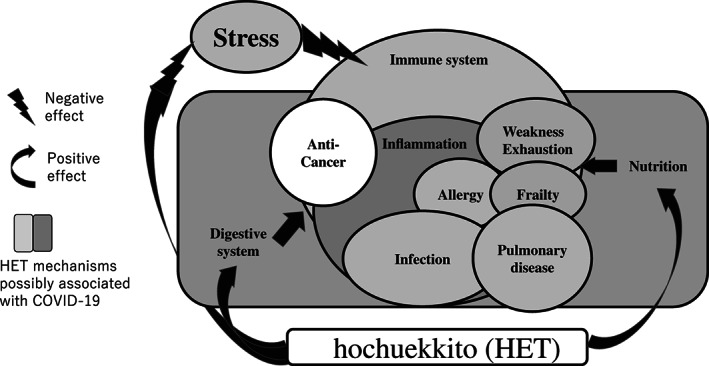
Schema of the efficacy of HET according to experimental and clinical studies for various conditions. Hochuekkito (HET: the Kampo extract formulation of hochuekkito, not decoction) described in this article is a drug with various complex effects that not only have treatment and prophylactic effects but improve the harmful effect of mental stress on the body.

Figure 3 shows a schema of the efficacy of HET via the immune system for various diseases and conditions, according to experimental and clinical studies. We can see that the immune system is related to the digestive system and nutrition.

Kampo medicines, which are composed of complex ingredients, may stimulate or suppress immunologically, depending on the condition. Immune‐stimulatory effects are defined as immunologically increasing values of the immune system, and immuno‐suppressive effects are defined as immunologically decreasing values of the immune system. In this article, from the standpoint of the treatment of disease, when the effects are thought to be favorable, they are collectively expressed as immune modulation.

Animal studies have shown that HET has the potential to stimulate immunity, not only in various models of immunosuppression but also in normal animals [18, 19].

HET was shown to be effective in the immune‐modulation of inflammation by various disease models [53, 54, 55, 56, 57, 58, 59, 60, 61, 62, 63, 64, 65]. Inflammation is related to infection, cancer, allergy, and other disorders. In these animal models, HET was shown to modulate innate immunity. Many kinds of cells and cytokines related to the immune system contribute to suppressing infection. Innate immunity is an immune response that eliminates foreign substances such as invading bacteria and viruses. It induces phagocytosis and inflammatory response. HET has an effect on innate immunity as shown by increases in NK activity and cytokines. In infection models of bacterial [23, 26, 27, 28, 29, 30] and viral infection [31, 32, 33, 34, 35, 36], HET also increased cytokines. The effects on various cytokines are recognized for the whole body, notably the lung and alveoli (Fig. 2). An example of other defense mechanisms against infection is that mucosal epithelial cells first capture pathogens in the respiratory tract. Alveolar macrophages also act as a homeostatic mechanism that produces inhibitory cytokines and protects against lung epithelial damage due to excessive inflammation.

In allergy, HET has been shown to modulate acquired immunity, modulating immune reaction on cytokines with HET results in improving both innate and acquired immunity to normal conditions.

To support immune function, nutrition, including the intake of calories, proteins, and vitamins, is very important [91]. Historically, the therapeutic target of hochuekkito was to improve the digestive system and nutritional problems. As an additional benefit, HET has been shown to secondarily boost immune function. Some of the traditional theory on which hochuekkito was based included supplementation of spleen [TM] and stomach [TM], which has been supported by recent studies showing modulation of the digestive and immune systems. The former is based on improving the response of the immune system to various conditions. The immune system can clearly be modulated or conditioned by food components and nutrients [91]. Improving nutrition and the general condition with HET may be one of the reasons for its ameliorating effects on exhaustion and frailty.

### Effect on weakness, exhaustion, frailty, and mental stress

Fatigue is caused by various factors, such as overwork, mental stress, and illness, including chronic inflammation. Fatigue is an important signal of disorders related to the homeostasis of the human body. Generally, when fatigue accumulates, people tend to get sick. In that respect, it is considered meaningful to improve the condition of fatigue in a preventive manner. There are some reports of HET improving fatigue, both from basic models [67, 68, 69] and clinical reports [86, 90]. In addition, at the time of acute viral infection, such as influenza, patients often experience general, long‐term fatigue. It is well known that inflammatory cytokines, including IL‐1β and/or antiviral cytokines including interferons (IFNs), are produced during these infections. In recent studies, peripherally produced cytokines have been shown to affect brain function [93, 94]. Cytokine expression in the brain is also thought to be involved in depressive symptoms. Viral infection can lead to prolonged cytokine elevation, and cytokine modulation with HET can contribute to improvement of this extreme cytokine reaction. It is noteworthy that HET has been shown to have the ability to aid in the recovery of corticosterone and the number and activity of some immunocompetent cells, such as macrophages and lymphocytes in the spleen [23, 25]. HET was developed considering the anti‐stress effects related to an extreme situation in ancient China, but it continues to be useful for dealing with the stress of fear and its subsequent depressive condition [70].

### The interaction between organ and viscera with HET

Recent studies have shown an interaction between the central brain and various organs or viscera, such as brain–gut, brain–heart, and brain–kidney, or organ and viscera, such as lung and kidney or gut and kidney [95, 96]. These interactions are transmitted through the autonomic nervous system, various neurotransmitters, hormones, cytokines, and even gut bacteria [96, 97, 98, 99, 100, 101]. Dysfunction of an interaction is caused by an imbalance in the complex autonomic nervous system, endocrine system, and immune system interactions. For example, stress‐induced corticotropin‐release factor irrigates gut function and causes abdominal pain or diarrhea [102]. This concept is well established in Kampo theory and has long been applied to diagnosis and treatment. A similar concept has been proven in recent experimental studies. HET has been shown in both basic and clinical research to be very effective for sub‐acute/chronic skin disease, such as in models of or patients with atopic dermatitis and inflammation of the skin [45, 46] and delayed healing of the skin [84]. In Kampo theory, the pulmonary [TM] system is directly relevant, not only the lung and airways but also the nose and skin surface [103]. The functions of the pulmonary [TM] system are to control respiration, water distribution, and defensive immune reaction against external pathogens. Because the lungs are immune‐rich, it is necessary to adjust immunity for diseases related to the pulmonary [TM] system. Because HET has an immune‐modulative effect, it is thought to be effective against skin diseases caused by dysregulation of the immune system.

### Application of HET to COVID‐19‐related prevention, treatment, and recovery

Worldwide, more than 15 million cases of COVID‐19 have been identified and more than 500 000 deaths have been recorded. In Japan, over 27 000 cases of confirmed COVID‐19 infection and over 980 deaths have been reported as of July 23rd of 2020. The clinical symptoms of COVID‐19 include fever, cough, and shortness of breath. The rapid development of pneumonia and respiratory failure can lead to a critical situation. In China, 81% of diagnosed cases were classified as mild, 14% as severe, and 5% as critical, and the overall case fatality rate was 2.3% [104]. Many studies of drugs are being conducted to investigate their efficacy for COVID‐19, but much is still unknown or controversial [1]. An important characteristic of COVID‐19 is that an infected person can infect other people about 48 h before the onset of symptoms, a key factor contributing to the spread of infection by asymptomatic carriers [1]. Development of vaccines and standard preventive measures such as hand washing and wearing a mask are important, but agents that can prevent the spread of infection need to be developed and introduced.

In the present study we summarized the immune‐stimulative and ‐modulative effects of HET on inflammatory conditions, the concept of nutritional support for the immune system, and recovery from weakness, exhaustion, frailty, and mental stress. From a long‐term perspective, HET's contribution to the maintenance of nutrition and support for the immune system would make it an ideal candidate for use in the prevention of SARS‐CoV‐2 infection.

In the present study, we introduced studies showing that HET improves systemic inflammation, nutrition, reduces the symptoms, decreases the number of common colds, and reduces exacerbation of COPD patients. In the same way and because of its immune‐regulative effect on the nose and skin surface, HET might also be effective for preventing the pneumonia caused by severe acute respiratory syndrome coronavirus 2 (SARS‐CoV‐2). Similarly, the same effect would be expected for elderly patients with complications such as diabetes and heart disease who are at significant risk for COVID‐19.

The pathophysiology of the cytokine release syndrome in severe cases is characterized by the dysregulation of immune response to pathogens, leading to cytokine overexpression. Because of its immune‐stimulative and modulative effect on inflammatory conditions, HET may be effective in reducing the amount of cytokine released, which is often a problem in severe cases of COVID‐19.

Prolonged self‐restraint affects the mind and body. Anxiety about infection can cause long‐lasting stress. Staying home for a long period can also lead to inactivity, which can cause a person to become frail. HET can be applied to improve the effects of anxiety and frailty caused by COVID‐19. One of the problems of recovery from the severe stage is vascular complications and decreased organ function. HET may be also useful for preventing inflammatory complications during rehabilitation for severe sequelae of cerebrovascular disease [82].

### Limitations

Evidence‐based studies remain insufficient to elucidate the effect of HET, thus further study will be necessary to establish its efficacy. Modern drugs can be tested by *in vitro* experiments using cell lines. However, even simple *in vitro* experimental results using HET directly on cells cannot be done because Kampo medicines (including HET) are only effective after oral administration is followed by metabolism by intestinal microbes and the liver. With the current scientific methods, basic research on the mechanism(s) of HET can prove only some of its clinical effects. We are hopeful that future research based on verification by the development of the Computational Modeling of the Human System, including systems biology, will clarify the utility of Kampo drugs in modern medicine.

## Conclusions

We examined the basic effects of HET in animal and clinical studies through an extensive literature search. We report that scientific investigations have shown that HET is highly effective against immune‐related diseases and conditions. Considering the effects on innate and acquired immunity, HET may have preventive and therapeutic effects on COVID‐19. Clinical trials will be necessary to verify and extend these reports on HET to provide clear evidence of its effectiveness.

## CONFLICTS OF INTEREST

TS and KA are members of the Department of Kampo and Integrative Medicine, Tohoku University Graduate School of Medicine, which does research in collaboration with Tsumura & Co. (Tokyo, Japan). MT, KM, and NT have received grant support from Tsumura & Co. TM received grant support from Kracie Pharmaceuticals and JPS Pharmaceuticals.
